# *In Situ* Hypothermic Perfusion in Hepatectomy for Advanced Hepatoblastoma: A Case Report and Literature Review

**DOI:** 10.70352/scrj.cr.25-0103

**Published:** 2025-09-30

**Authors:** Toshiharu Matsuura, Yusuke Yanagi, Naonori Kawakubo, Keisuke Kajihara, Takeshi Shirai, Yasuyuki Uchida, Yukihiro Toriigahara, Tomoharu Yoshizumi, Tatsuro Tajiri

**Affiliations:** 1 Department of Pediatric Surgery, Graduate School of Medical Sciences, Kyushu University, Fukuoka, Fukuoka, Japan; 2 Department of Surgery and Science, Graduate School of Medical Sciences, Kyushu University, Fukuoka, Fukuoka, Japan

**Keywords:** hepatoblastoma, *in situ* hypothermic perfusion, *ante situm*, extreme liver resection

## Abstract

**INTRODUCTION:**

Hepatoblastoma with tumor thrombi involving three hepatic veins or extending into the inferior vena cava (IVC) toward the right atrium is mostly considered unresectable. In the current era of liver transplantation for unresectable hepatoblastoma with stable outcomes, it is challenging to choose extreme hepatectomy with hypothermic perfusion under total hepatic vascular exclusion. We herein report a successful case of extreme liver resection with *in situ* hypothermic perfusion.

**CASE PRESENTATION:**

A 10-month-old girl was referred for an unresectable hepatoblastoma (alpha-fetoprotein [AFP] 592037 ng/mL), approximately 12 cm in size, occupying the right lobe with a tumoral thrombus extending into the IVC toward the right atrium. The tumor continued to involve three hepatic veins and the IVC after intensive chemotherapy, which was categorized into POSTTEXT III (P1, V3, M0) staging. We planned an *ante situm* technique or *in situ* hypothermic perfusion to accomplish the complete removal of the large tumor and venous reconstruction. The adopted surgical technique was as follows: (1) hepatic partition on the line of extended right hepatectomy, including segment Iva; (2) mobilization of the suprahepatic IVC with tumor thrombi; (3) insertion of a cannula for hypothermic perfusion was from the stump of the right portal vein to the left lobe; (4) complete occlusion of the hepatic inflow following systemic heparinization with cutting the left hepatic vein and hypothermic perfusion with cold preservation solution using crushed ice; (5) complete tumor removal with the IVC tumoral thrombi; and (6) reconstruction of the IVC and left hepatic vein using an artificial vessel graft without requiring *ante situm* position, followed by reperfusion of the remnant liver. Time for cooling and preservation of the remaining liver in the body was 40 minutes, and time for IVC reconstruction was 21 minutes. The histological examination was margin-negative, and the AFP level was normalized.

**CONCLUSIONS:**

Resection of liver tumors invading the IVC or hepatic veins has become possible with technical lessons learned from liver transplantation skills. This procedure is a realistic option for achieving surgical cure and improving the quality of life in select pediatric patients with otherwise unresectable hepatoblastomas.

## Abbreviations

AFP
: alpha-fetoprotein
AST
: aspartate transaminase
ALT
: alanine aminotransferase
CPB
: cardiopulmonary bypass
ePTFE
: expanded polytetrafluoroethylene
HB
: hepatoblastoma
HTK
: histidine-tryptophan-ketoglutarate
HV
: hepatic vein
ICG
: indocyanine green
IVC
: inferior vena cava
LHV
: left hepatic vein
LMW
: low-molecular-weight
MHV
: middle hepatic vein
NA
: not available
PV
: portal vein
RHV
: right hepatic vein
T. Bil
: total bilirubin
THVE
: total hepatic vascular exclusion
UW
: University of Wisconsin
VVBP
: veno-venous bypass
WF
: warfarin

## INTRODUCTION

Over several decades, various liver resection techniques for liver malignancies have been developed and modified to achieve curative resection with negative margins. Especially for conventionally unresectable tumors, such as those invading the hepatic hilum, inferior vena cava (IVC), or hepatic veins (HV), technically challenging liver surgeries using partial or total hepatic vascular exclusion (THVE) are required.

In 1971, Fortner et al.^1)^ first reported experimental studies in canine models and the clinical application of *in situ* hypothermic perfusion and liver resection for tumors deemed irresectable. In 1988, Pichlmayr et al.^2)^ first introduced total hepatectomy for resecting liver tumors *ex situ* with subsequent orthotopic autotransplantation of the liver remnant. Thereafter, Hannoun et al.^3)^ in 1991 first reported a modified type of surgery with an *ante situm* approach, where the liver remains *in situ* with THVE and undergoes *in situ* cold preservation during the outflow vascular reconstruction under the ventral rotation (*ante situm* position) of the whole liver achieved by dividing the suprahepatic IVC or HVs. While the techniques of these complex liver resection procedures have developed, liver transplantation, initiated in 1963, has also seen dramatical improvements in its outcomes and become accepted worldwide.

Hepatoblastoma (HB) is the most common pediatric malignant liver tumor, occurring in the first 3 years of life.^4)^ The optimal surgical concept for HB is “complete resection” and liver transplantation is applied for “unresectable” HB cases. However, the definition of irresectability may depend on whether the center has experienced advanced liver resection using THVE and liver transplantation.

We herein report a case in which HB deemed unresectable by conventional resection was successfully resected using *in situ* hypothermic perfusion of the remnant liver under THVE.

## CASE PRESENTATION

**Fig. 1:**
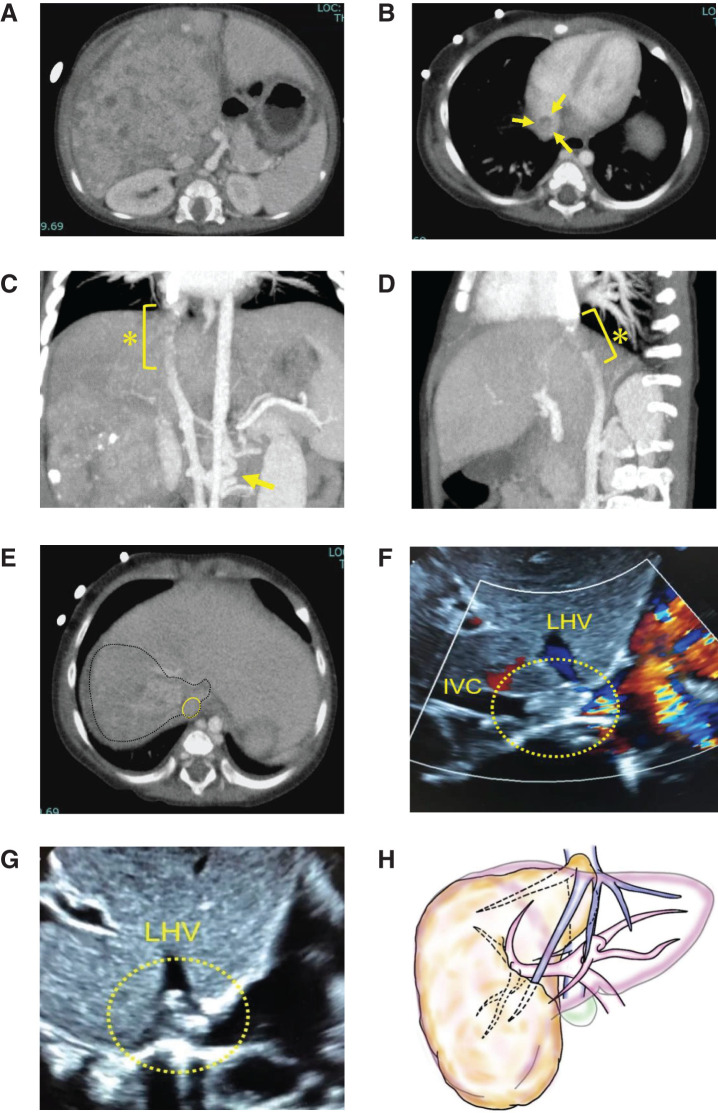
(**A**) CT revealed a 12-cm hepatic tumor located predominantly in the right lobe. (**B**) CT scan showing hepatoblastoma with tumor thrombus (arrows) into the IVC close to the right atrium. (**C**, **D**) The extent of tumor thrombus in IVC (asterisk) on coronal and sagittal plane CT scan, respectively. The tumor completely occupied from the retrohepatic IVC to the slightly above level of the diaphragm. The developed collateral vein (arrow in [**C**]) drained into the azygous vein. (**E**) Relation of the tumor (encircled by black dot line) and the hepatic veins (yellow circle indicated IVC). The tumor had also completely infiltrated the confluence of the LHV. (**F**, **G**) The tumor thrombi (yellow dot circle) were confirmed at the confluence of the LHV and IVC by Doppler ultrasound. (**H**) Hepatoblastoma occupied the entire right lobe and a portion of segment IVa. The tumor invaded into the IVC toward the right atrium, staged as POSTTEXT III (P1, V3, M0). IVC, inferior vena cava; LHV, left hepatic vein

A 10-month-old Japanese girl was referred for a large palpable mass in the right upper quadrant. CT revealed a 12-cm hepatic tumor located predominantly in the right lobe (**Fig. 1A**). No distant metastasis was found, but the tumor involved the right (RHV), middle (MHV), and confluence of the left hepatic vein (LHV), and further invaded the IVC, obstructing it near the right atrium. The serum alpha-fetoprotein (AFP) level was 592037 ng/mL, and the tumor was diagnosed as HB, as proven by a biopsy.

The patient received preoperative chemotherapy with two cycles of cisplatin, vincristine, adriamycin, and cyclophosphamide followed by one cycle of carboplatin, ifosfamide, and etoposide. Although the serum AFP level (1486 ng/mL) was remarkably decreased after chemotherapy, heart color Doppler ultrasound revealed tumor thrombus in three hepatic veins and IVC close to the right atrium (**Fig. 1B**, **1F**, **1G**) and was categorized as POSTTEXT III (P1, V3, M0) staging. According to the preoperative CT images, the tumor thrombus seemed to occlude the IVC completely and invade the LHV including V4 (**Fig. 1C**–**1E**). The collateral veins were developed and drained into the dilated azygous vein because of the IVC obstruction by tumor thrombus (**Fig. 1C**). Our surgical team planned an extended right lobe and a portion of segment IVa hepatectomy combined with vascular resection of the thrombosed IVC and LHV, and reconstruction under *in situ* cold preservation of the remnant liver.

### Surgical technique

The abdomen was explored using a transverse incision in the upper abdomen. There was no evidence of ascites or peritoneal metastasis. The location and extent of the hepatic tumors were examined using intraoperative ultrasonography. A tumor thrombus was identified in the IVC and extended into the confluence of the MHV and LHV, similar to preoperative imaging. In addition, we also confirmed that the tumor was luminescent by indocyanine green (ICG) injected (0.5 mg/kg) 3 days prior to surgery.

The procedure was initiated by mobilizing the liver, followed by cholecystectomy. During cholecystectomy, a 4-Fr cannula for intraoperative cholangiography was inserted through the cystic duct. The right hepatic artery was ligated and divided. The anterior and posterior branches of the right portal vein (PV) were dissected as long as possible so that we could later insert the cannula for the perfusate into the left lobe through the right PV stump. Although the right lobe was not completely able to mobilize from the IVC because of tumor invasion to the IVC, in order to prepare for the *ante situm* position, the left lobe, including Spiegel’s portion of the caudate lobe, was fully mobilized from the surrounding ligaments and IVC.

The hepatic partition with CUSA using the Pringle maneuver was started on the demarcation line of the conventional extended right hepatectomy toward segment IVa and finally transected to a plane that would divide the intrahepatic LHV thrombosed with the tumor. Because the top of the tumor thrombus in the IVC was located at the level of the diaphragm, the suprahepatic IVC was completely detached from the diaphragmatic hiatus by bilateral phrenic vein ligation so that the IVC could be pulled into the abdominal cavity to clamp as far apart from the tumor as possible. The IVC was completely mobilized from the retroperitoneum between the diaphragm hiatus and level of the right renal vein. After confirming a safe transection site using cholangiography, the right bile duct was ligated and divided. After the anterior and posterior branches of the right PV were divided, a 10-Fr Large Flow cannula (Senko Medical Instrument Mfg. Co., Ltd., Tokyo, Japan) was inserted and fixed through the stump of the right PV to prepare for hypothermic perfusion of the left lobe of the liver. After systemic heparinization (40 U/kg, intravenously) followed by 2 minutes of waiting, the left hepatic artery and bile ducts, including the main trunk of the PV, were bulk-clamped using the Pringle maneuver, followed by a total clamp of the infrahepatic and suprahepatic vena cava, respectively. The anhepatic phase was initiated until the remnant left lobe reperfusion.

**Fig. 2:**
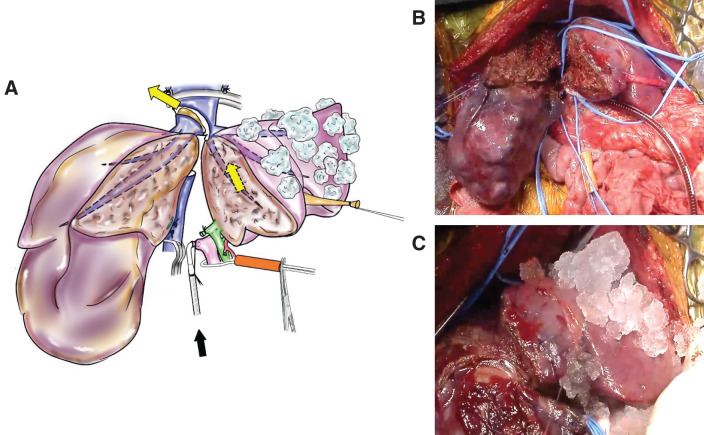
(**A**) The intrahepatic LHV was opened for venting and then *in situ* hypothermic perfusion was initiated with 1000 mL of 4°C HTK solution via the inserted PV cannula (black arrow) and washing out of the left lobe (yellow arrow). In addition, crushed sterile ice was placed around the left lobe to help cool the liver. (**B**) Liver partition was performed, and the PV was canulated for *in situ* hypothermic perfusion. (**C**) Crushed sterile ice was placed around the left lobe to help cool the left lobe. HTK, histidine-tryptophan-ketoglutarate; LHV, left hepatic vein; PV, portal vein

The intrahepatic LHV at the distal level of the tumor thrombus was opened for venting and then *in situ* hypothermic perfusion of the left lobe was initiated with 1000 mL of histidine-tryptophan-ketoglutarate (HTK) preservation solution (Custodiol; Essential Pharmaceuticals, Durham, NC, USA) cooled to 4°C via the PV cannula to minimize ischemic injury to the left lobe (**Fig. 2**). We selected the HTK solution as a perfusate because of its low viscosity and low potassium content as the electrolyte composition. In addition, crushed sterile ice was placed around the left lobe to cool the liver. The outflow of cold perfusate from the opened LHV was excellent. The tumor exposed in the open LHV and IVC was carefully resected in a bloodless field. The infrahepatic IVC was completely divided because the tumor invaded and adhered to the entire circumference of the IVC and could not be pull out, and the suprahepatic IVC was almost divided, except for the lateral wall, which was barely connected with that of the LHV. The specimen was completely removed, and there was no remnant tumor in the residual liver or IVC wall, as confirmed using the ICG luminescence camera. As a result, because there was no need to completely disconnect the IVC and LHV, the *ante situm* position was not applied.

**Fig. 3:**
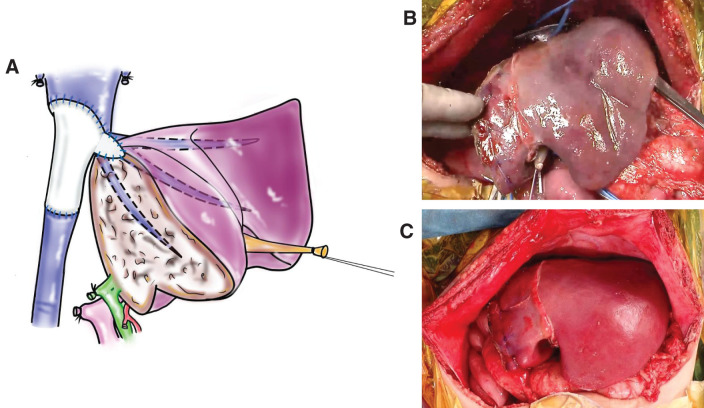
(**A**) The IVC was completely interposed covering the defect of the LHV with an 8-mm-diameter ringed ePTFE artificial vessel graft, and then the left lobe was re-perfused. (**B**) The appearance of the liver during *in situ* hypothermic perfusion. (**C**) The gross coloration of the left lobe became good again after reperfusion. ePTFE, expanded polytetrafluoroethylene; IVC, inferior vena cava; LHV, left hepatic vein

The IVC was completely interposed with an 8-mm-diameter expanded polytetrafluoroethylene (ePTFE) artificial vessel graft (W.L. Gore & Associates, Tokyo, Japan). The suprahepatic IVC and LHV were anastomosed with the artificial vessel graft cut diagonally by 5–0 PDS continuous sutures, followed by end-to-end anastomosis with the infrahepatic IVC. When IVC reconstruction was complete, hypothermic perfusion was discontinued. The suprahepatic and infrahepatic IVC clamps were removed, and the remnant left lobe was reperfused by releasing the Pringle clamp. The gross coloration of the left lobe improved again, and the blood inflow and outflow of the remnant liver were both sufficient, as confirmed by intraoperative ultrasonography (**Fig. 3**). The total anhepatic time was 41 minutes, and the *in situ* hypothermic perfusion time of the left lobe was 40 minutes, including 21 minutes for IVC reconstruction.

**Fig. 4:**
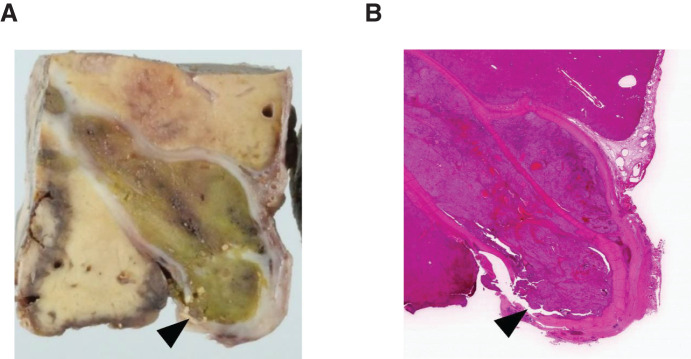
(**A**, **B**) The macro- and microscopical histopathological findings of the removed tumor. The pathological diagnosis was a mixed type of hepatoblastoma invading the hepatic veins and IVC extensively, but the surgical margins were negative. The tumor thrombus in IVC (arrowhead) had infiltrated the entire circumference and tightly adhered to the vessel wall. IVC, inferior vena cava

The resected liver weighed 320 g. The histological diagnosis of the tumor was a mixed type of HB invading the hepatic veins and IVC extensively, but the surgical margins were negative (**Fig. 4**).

### Postoperative course

**Fig. 5:**
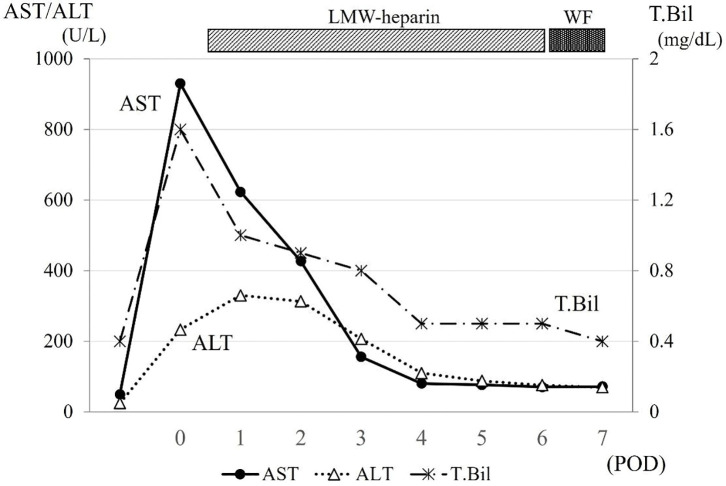
Postoperative course of transaminase and bilirubin levels. Serum transaminase values were elevated on the day following surgery (both <1000 IU/L) and decreased rapidly. Total bilirubin levels showed a similar course to serum transaminase. ALT, alanine aminotransferase; AST, aspartate transaminase; LMW, low-molecular-weight; POD, post operative day; T. Bil, total bilirubin; WF, warfarin

**Fig. 6:**
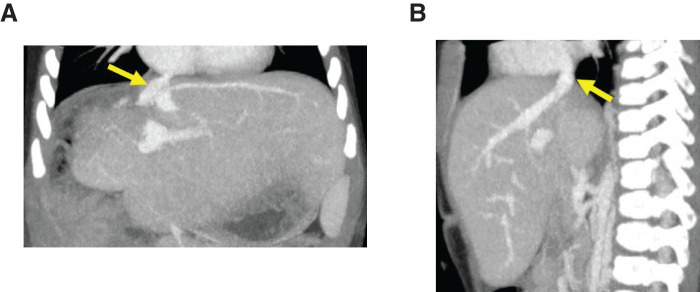
(**A**, **B**) Contrast-enhanced CT scan with coronal and sagittal plane showed the absence of tumor recurrence and patency of the left hepatic vein reconstructed by the ePTFE graft (arrow). ePTFE, expanded polytetrafluoroethylene

Although serum transaminases levels were elevated on the day following surgery (AST, 933 U/L; ALT, 233 U/L), they immediately decreased to the normal range within a week (**Fig. 5**). The bilirubin levels remained within the normal range throughout the postoperative course. Due to the use of an artificial vessel graft, anticoagulation therapy was introduced postoperatively, starting with low-molecular-weight heparin and maintained with warfarin targeting PT-INR around 1.5–2.0. Two weeks after surgery, without any surgical complications, adjuvant chemotherapy was initiated, consisting of vincristine and irinotecan for two cycles. Two years after the completion of any treatment, although the venous flow at retrohepatic IVC replaced by the ePTFE graft was very low due to the steal phenomenon to the dilated azygous vein that had developed preoperatively, contrast-enhanced CT showed the absence of tumor recurrence and patency of the left hepatic vein reconstructed by the ePTFE graft (**Fig. 6**). The patient is currently showing a good clinical course with a normal liver function and a normal AFP level (2.1 ng/mL).

## DISCUSSION

The current treatment strategies and surgical resectability of HB depend on the PRETEXT and POSTTEXT staging systems. Approximately 60% of HB cases are unresectable at referral,^4)^ but this tumor is highly chemo-sensitive, and 50%–85% of cases become resectable after cisplatin-based neoadjuvant chemotherapy.^5,6)^ Complete surgical removal of HB by liver resection or transplantation is the only curative treatment that can achieve a long-term survival. Liver resection is generally indicated for tumors that are able to spare at least one branch of the PV and one HV draining the area.

However, the definition of “irresectability” remains unclear, and its definition is getting more complex with advances in surgical techniques and devices. We should carefully consider whether or not a patient with unfortunate tumor localization invading the IVC or three HVs should be excluded from surgical resection. Although liver transplantation is more likely to achieve complete tumor removal than liver resection, the latter avoids exposing young patients to long-term immunosuppression.

Extreme liver resection using *ex situ* liver resection was first described by Pichlmayr et al.^2)^ in 1988 and consisted of total hepatectomy to resect large tumors *ex situ* with subsequent orthotopic autotransplantation of the remnant liver. Historical data of *ex situ* procedures showed an unacceptable-high mortality rate of over 30%, mainly due to an impaired liver function and sepsis caused by the prolonged duration of surgery and anhepatic phase.^7)^ A recent meta-analysis of *ex situ* procedures revealed a lower mortality rate (11.6%),^8)^ but the additional risk of complications related to arterial and biliary reconstruction in autotransplantation is unavoidable. The *ante situm* liver resection introduced by Hannoun et al.^3)^ in 1991 is characterized by no hepatoduodenal ligament division, *in situ* cold liver perfusion with THVE, and division of the suprahepatic IVC, which allows the ventral rotation of the liver (*ante situm* position), thereby providing surgeons with excellent visualization of the HVs confluence and retrohepatic IVC. A review of hypothermic *ante situm* resection in hepatocaval confluence tumors suggests that this approach is feasible and safer than the *ex situ* procedure, with an acceptable morbidity and mortality rate.^9)^ The final decision concerning the need to use the *ante situm* procedure can only be made intraoperatively, as some tumors eventually peeled off, or a part of the IVC wall could be preserved without the need for transection, as in our case.

Organ preservation and vascular reconstruction techniques are highly specialized skills unique to transplant surgery. There have been limited reports of extreme liver resection for advanced HB using the surgical skills of transplantation (**Table 1**).^10–12)^ There was one case treated with *ex situ* and autotransplantation, three cases managed with *ante situm*, and our case managed with *in situ*. In most cases, the University of Wisconsin (UW) or HTK solution is commonly used in liver transplantation as an organ hypothermic perfusate. When using cold perfusate *in situ*, HTK solution is preferred to UW solution to avoid unexpected inflow to the systemic circulation, as the UW solution has high viscosity and high potassium as its electrolyte composition. Two cases with tumoral thrombi extending the IVC into the right atrium and complete removal of the tumor in the right atrium were accomplished through cardiopulmonary bypass (CPB) at low cardiac arrest. In another two cases, veno-venous bypass (VVBP) was additionally used to control intraoperative bleeding and prevent intestinal congestion during portal venous clamping. Although VVBP is recommended in patients requiring complex hepatectomy under THVE exceeding 60–90 minutes,^13)^ our case was tolerable without applying VVBP during venous reconstruction. During surgery, it was determined that the time for THVE could be shortened by initiating hypothermic perfusion after liver transection. Dubay et al.^14)^ also showed that *in situ* hypothermic perfusion could be performed without VVBP. When a short reconstruction time is anticipated, VVBP can be omitted with close monitoring of vital and intestinal congestion.

**Table 1:** Extreme liver resection using transplant surgical techniques in hepatoblastoma

Case	Report	Age (yrs)	Vascular infiltration	Type of resection	CPB or VVBP	Cold storage solution	Vessel graft for reconstruction	Hypothermic time (min)	Outcome
1	Shi et al. (2018)^10)^	1	Right atrium, IVC	*Ex situ* with autotransplantation	CPB	UW	None	190	Alive
2	Angelico et al. (2017)^11)^	0	Right atrium, IVC	*Ante situm*	CPB	Celsior	Deceased donor aortic graft for IVC	71	Alive
3	Schlegel et al. (2020)^12)^	3	≥2 HVs, IVC	*Ante situm*	VVBP	HTK	Deceased donor vein graft for IVC	NA	Alive
4		8	≥2 HVs, IVC	*Ante situm*	VVBP	Normal saline	Deceased donor vein graft for IVC	NA	Alive
5	Current case	0	3 HVs, IVC	*In situ*	No	HTK	ePTFE graft for IVC	40	Alive

CPB, cardiopulmonary bypass; ePTFE, expanded polytetrafluoroethylene; HTK, histidine-tryptophan-ketoglutarate; HV, hepatic vein; IVC, inferior vena cava; NA, not available; UW, University of Wisconsin; VVBP, veno-venous bypass

Deceased donor vessel grafts were used for IVC reconstruction in three previous cases, as summarized in **Table 1**. However, in Japan, deceased donors are limited, and cryopreserved vessels are not available. In addition, it is extremely difficult to obtain sufficient autologous vessels from infants to replace the IVC, both in size and length. Therefore, we used a ringed ePTFE graft with an appropriate size to interpose the removed IVC. The use of artificial vessels for pediatric cases is concerned that they would be stenotic as the infant grows. However, in the present case, the IVC flow was completely occluded preoperatively by tumor thrombus and the azygous circulation developed. Therefore, we believed that the venous circulation would be maintained via the azygous vein even if the artificial vessel replaced IVC became stenotic in the future.

Although one potential disadvantage of using prosthetic materials is the risk of infection, an ePTFE graft is thought to be quite resistant to infection compared with other types of prosthetic grafts and has recently been used in common, even in living donor liver transplantation for reconstruction of V5 and V8 outflow in right lobe grafts.^15)^ We used low-molecular-weight heparin switched to warfarin for maintenance, but whether or not long-term anticoagulation reduces long-term complications remains questionable. Future study is warranted to establish guidelines because of the lack of high-level evidence in the literature on this clinical question.

In the current era of liver transplantation for HB with stable outcomes, it is difficult to determine whether or not the challenging approach of extreme hepatectomy under THVE should be attempted. The use of *in situ* hypothermic liver resection can be encouraged for patients whose tumor invasion localized at the hepatocaval confluence of three hepatic veins, including the IVC. The vascular reconstruction under prolonged vascular clamping is feasible using organ preservation techniques of liver transplantation. On the contrary, the application of this technique for those patients who have received preoperative heavy chemotherapy should be considered carefully, in the light of a reported postoperative liver failure death following the use of hypothermic liver perfusion in a patient with severe steatohepatitis after intensive preoperative chemotherapy.^14)^ Extreme liver resection such as *ex situ*, *ante situm*, and *in situ* should be performed only in very select cases and, depending on the expected surgical risk, under the backup of living donors in an experienced pediatric liver transplant center.

## CONCLUSIONS

Resection of liver tumors invading the IVC or HVs has become possible with technical lessons learned from liver transplantation procedures. Therefore, extreme liver resection and vascular reconstruction under THVE requires a specialized center where surgeons familiar with both complex hepatobiliary surgery and pediatric liver transplantation are available. This procedure is a realistic option for achieving a surgical cure and improved quality of life in selected pediatric patients with unresectable tumors.
